# The Use of Telehealth Among People Living With Dementia-Caregiver Dyads During the COVID-19 Pandemic: Scoping Review

**DOI:** 10.2196/45045

**Published:** 2023-05-25

**Authors:** Jiaming Liang, Maria P Aranda

**Affiliations:** 1 Edward R Roybal Institute on Aging Suzanne Dworak-Peck School of Social Work University of Southern California Los Angeles, CA United States

**Keywords:** scoping review, COVID-19, telehealth, people living with dementia, family caregiver, mobile phone

## Abstract

**Background:**

Telehealth has gained substantial attention during the COVID-19 pandemic, and reimbursement policies in health care settings have increased access to remote modes of care delivery. Telehealth has the potential to mitigate care concerns for people living with dementia and their family caregivers. There is a paucity of knowledge on the performance of telehealth services and user experiences, especially among caregiving dyads during the pandemic.

**Objective:**

This study aims to describe the implementation, effectiveness, user experience, and barriers to accessing and using telehealth services for people living with dementia and their caregivers during the COVID-19 pandemic.

**Methods:**

Following the PRISMA-ScR (Preferred Reporting Items for Systematic Reviews and Meta-Analyses extension for Scoping Reviews) checklist, we searched 7 databases (PubMed, PsycINFO, AgeLine, CINAHL, Social Services Abstracts, Web of Science, and Scopus) and a web-based search engine (Google Scholar). The inclusion criteria for peer-reviewed English publications from March 2020 to August 2022 consisted of studies related to telehealth services for people living with dementia and their family caregivers and studies conducted during the COVID-19 pandemic.

**Results:**

A total of 24 articles (10 quantitative and 14 qualitative studies) from 10 different countries were included. The major findings of the reviewed articles were extracted and organized into the following 4 themes: study design characteristics—strategies were adopted to improve the accessibility and experience of people living with dementia-caregiver dyads; efficacy outcomes of telehealth services—robust evidence is lacking on the comparative effectiveness of in-person services; perceived experiences of people living with dementia and caregivers—most reviewed studies reported positive experiences of using telehealth services and perceived personal and social benefits from their participants; and barriers to accessing and using telehealth services—several barriers related to individuals, infrastructure, and telehealth environments were identified.

**Conclusions:**

Although evidence of its effectiveness is still limited, telehealth is widely accepted as a viable alternative to in-person care for high-risk groups, such as people living with dementia and their caregivers. Future research should include expanding digital access for those with limited resources and low technology literacy, adopting randomized controlled trial designs to establish the comparative effectiveness of different modes of service delivery, and increasing the sample diversity.

## Introduction

### Background

Since the first case of COVID-19 was reported in China in late 2019, public health systems around the world have encountered unprecedented challenges. In response to the global pandemic, countries have actively taken preventive measures, such as banning large gatherings, stopping the acceptance of visitors from certain countries, and restricting in-person health care [1]. Older people living with dementia and their families were disproportionately affected by the pandemic. A study found that from March to December 2020, the mortality rate of people living with dementia increased by 25.7% compared with the same period during the previous year, which was almost double the rate of the older population with no dementia [2]. Pandemic-induced social isolation and reduced health care services also exacerbated behavioral and psychiatric symptoms of people living with dementia and increased care burden and psychological distress for family caregivers [3-5].

### Challenges for People Living With Dementia and Caregivers

Although countries took drastic measures to combat the virus, people living with dementia and their family caregivers faced numerous challenges. First, people living with dementia often have comorbid medical conditions such that foregoing health care appointments and scheduled inpatient admissions can seriously affect their health status and prognosis [6,7]. Second, many people living with advanced dementia may not be able to understand what a pandemic is, how the virus can harm their health, or why they need to stay in isolation for weeks at a time. These situations can increase susceptibility to neuropsychiatric reactions, including depression, anxiety, and agitation [8-10]. Third, in-home isolation or quarantine limits the range of physical, cognitive, and social activities, which may worsen executive function, memory, mobility, and managing daily activities [10-12]. Such challenges add to the responsibilities and care concerns of family caregivers who are left to make unprepared medical decisions. Furthermore, with the lack of access to support services, such as respite care and in-home care, caregivers stand to lose time that they may rely on for their own self-care [13] or face disruptions to at-home employment activities that increased during the pandemic [14].

### Telehealth as a Potential Solution

Telehealth primarily refers to connecting health care providers and accessing relevant services via an internet-connected computer, tablet, or smartphone. It can be categorized as synchronous, with real-time communication between the provider and user, or asynchronous, with delayed communication through data, images, messages, or prerecorded videos [15]. Telehealth is rapidly becoming the primary mode of health care delivery in many countries because of the pandemic [16]. Even before COVID-19, many services were already available through telehealth for clinical consultation, diagnosis, evaluation, and psychotherapy [17,18]. For people living with dementia and their caregivers, early studies indicated that video telehealth consultation can help clinicians make timely diagnoses, especially for those living in rural areas [19]. A review found that home video telehealth was effective in addressing caregivers’ psychological concerns [20].

Studying the phenomenon of telehealth is timely. During the pandemic, many outdoor activities were restricted, and telehealth became the only choice for most people to take care of their health care needs. This was coupled with the fact that insurers were more likely to cover telehealth methods for their members’ health care encounters [21]. Thus, these exigent conditions enabled the natural experiment to study the impact of telehealth during the pandemic, as the influence of personal preferences decreased to a large extent because of public health mandates and abrupt changes in insurers covering telehealth to the degree witnessed during the pandemic. Moreover, compared with the *timing* of previous global pandemics, such as severe acute respiratory syndrome in 2003 [22], today’s world is more technologically advanced with faster internet and smarter mobile devices that allow users to access telehealth services easily and quickly [16]. However, we should also be aware that the digital divide may hinder people living with dementia from benefiting from technology. For example, the smartphone ownership rate among people aged ≥65 years is only 61%, which is significantly lower than the 90% for people aged 18 to 35 years [23]. Thus, it is imperative to explore the experience of and barriers to using telehealth services among people living with dementia and their caregivers.

### This Scoping Review

In sum, telehealth may help medical and social service providers deliver stable support to people living with dementia and their caregivers and help improve quality of life overall. To this end, we conducted a scoping review of the role of telehealth in the lives of people living with dementia and their family caregivers during the COVID-19 pandemic. In our review, we were interested in understanding what specific strategies were adopted to improve telehealth user accessibility and implementation success, what telehealth services people living with dementia and caregivers used, and what barriers existed while using these services. This information is needed to inform the knowledge base on the effectiveness of telehealth programs and the development of telehealth best practices and to enhance user experiences with telehealth services in the future.

## Methods

### Overview

This scoping review was conducted following the guidelines of the PRISMA-ScR (Preferred Reporting Items for Systematic Reviews and Meta-Analyses extension for Scoping Reviews) checklist [24]. The PRISMA-ScR checklist was developed based on the PRISMA (Preferred Reporting Items for Systematic Reviews and Meta-Analyses) statement, which provides guidelines for systematic reviews and meta-analyses by revising different essential reporting items for this specific type of knowledge synthesis [24].

### Search Strategy

An extensive search for articles from March 2020 to August 2022 was conducted by (first author) across 7 databases—PubMed, PsycINFO, AgeLine, CINAHL, Social Services Abstracts, Web of Science, and Scopus—and 1 web-based search engine, Google Scholar. Preprint servers were excluded from the search strategy because of their lack of peer review and quality control. Although they may provide valuable information, their findings may change during the peer-review process, raising concerns about the reliability and efficacy of relevant telehealth services. The search strategy was developed across the following four domains: (1) COVID-19, (2) people with cognitive decline, (3) family caregivers, and (4) telehealth. We identified relevant keywords and Medical Subject Headings of the 4 domains through an initial limited search on PubMed and used different combinations of keywords and Medical Subject Headings terms to search in databases (Multimedia Appendix 1 provides the detailed search strategy). The search on Google Scholar was limited to the top 200 results, which covered 5 pages after the last relevant article. For a comprehensive search, we reviewed the reference lists of all included articles to identify additional studies.

### Eligibility Criteria

Eligibility criteria were established for screening. First, we considered only English publications in peer-reviewed journals but did not limit publication types; thus, original research articles, brief reports, and reviews were included for screening. Second, the included studies must relate to telehealth services for community-dwelling older adults living with dementia or cognitive impairment, family caregivers who provide assistance to loved ones with dementia or cognitive impairment, or both groups. The study participants were not restricted to people with dementia or caregivers to include perspectives from telehealth service providers and other relevant stakeholders. Third, eligible studies could be quantitative, qualitative, or mixed method studies and must have been conducted during the COVID-19 pandemic. We excluded articles that focused on institutionalized older adults and articles that only mentioned the potential benefits of telehealth in the discussion section.

### Data Extraction and Analysis

All identified articles were imported into Covidence (Veritas Health Innovation), a web-based systematic review software, and duplicates were removed [25]. On the basis of inclusion criteria, the first author screened the titles and abstracts of the identified articles. If there was a lack of clarity on whether an article could be included in the review, then full-text screening was performed, and the final consensus was confirmed with the second author, a senior systematic review expert, who supervised the entire article selection process and ensured that all inclusion and exclusion criteria were correctly applied.

We then developed a standardized Microsoft Excel worksheet to extract information from the included studies, such as bibliographic references, country in which the study was conducted, research methodology, study aims, sample, service or intervention components, access considerations, and major findings.

The extracted data were analyzed using inductive and deductive thematic analyses [26]. Initially, a preliminary codebook comprising 4 major themes (ie, study design characteristics, program efficacy, participants’ experience, and relevant barriers) was developed in accordance with the research questions of this review. Next, both authors became familiar with the data and generated the initial codes. The generated codes were then revised and merged through an iterative process and eventually classified into 4 major themes, with each subtheme named accordingly. The classification and naming of each theme and its subthemes were agreed on by both the authors. Owing to the nascent stage of research on this topic, no research quality assessment tool was applied to the review.

### Ethics Approval

This paper is a scoping review that did not involve the use of human or animal subjects by any of the authors. As such, it is exempt from ethical approval requirements. The review was conducted in accordance with good scientific practices.

## Results

### Overview of the Included Studies

We initially identified 964 records in total (956 from the database search and 8 from the bibliography search). After removing 275 duplicates, title and abstract screening was performed on 689 records, and 546 were removed because they did not focus on people living with dementia, caregivers, or telehealth service providers (168/546, 30.8%), were not related to COVID-19 (78/546, 14.3%), were not on telehealth (32/546, 5.9%), were not in peer-reviewed journals (31/546, 5.7%), and did not meet 2 or more eligibility criteria (237/546, 43.4%).

**Figure 1:**
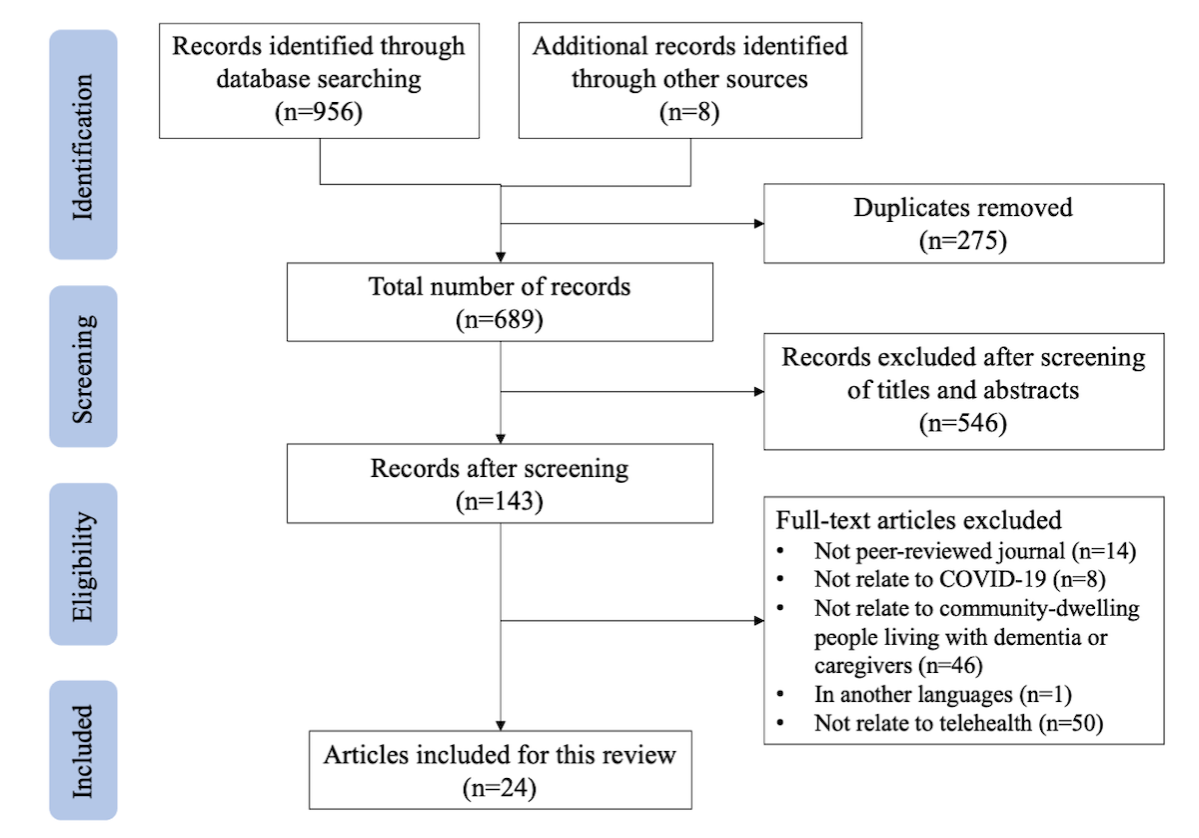
The PRISMA (Preferred Reporting Items for Systematic Reviews and Meta-Analyses) flow diagram of this study.

Of the 143 articles assessed in full text, 119 were excluded for failing to meet the eligibility criteria, including 14 articles from non–peer-reviewed sources, 8 studies unrelated to COVID-19 or conducted outside of the pandemic period, 46 studies not related to telehealth services for older people with dementia and caregivers, 1 article written in the Arabic language, and 50 studies unrelated to telehealth. Our sample of 24 articles were included for the final review and data extraction (Figure 1).

Table 1 provides the descriptive characteristics of all included articles. These 24 studies were from 9 different countries: 6 from the United States; 5 from the United Kingdom; 4 from Italy; and 9 from Argentina, Brazil, Canada, China, Ireland, New Zealand, and Spain. In total, 10 articles reported quantitative research results, and 14 articles reported qualitative research results. No mixed method studies were found. With regard to the study goal, 5 articles focused on the efficacy of telehealth services, 10 were pilot studies establishing the feasibility of providing or testing the interventions, and 9 interviewed participants or telehealth users to describe their experience or perceptions of the telehealth service or intervention (2 quantitative and 7 qualitative).

Telehealth services can be classified into 2 main categories: routine medical care services (n=6) and telehealth interventions (n=18). Routine medical care services are remote health care services provided by professional medical service organizations to individuals living with dementia or cognitive impairment. These services typically include medical consultation and prescription refills and so on. A total of 6 studies in this category focused on synchronous teleconsultation provided by neurologists and physicians, with 4 using a video-based approach and 2 offering both phone and video options [27-32].

Telehealth interventions refer to structured and time-limited cognitive, behavioral, and emotional therapies; rehabilitation; or training programs for people living with dementia, their caregivers, or both. Caregiving skills training and support group activities are also included in this category. Among the 18 telehealth intervention studies, 13 targeted people living with dementia, with 7 being one-on-one synchronous programs (2 on telephone only [33,34], 1 video-based only [35], and 4 providing mixed options [36-39]), 2 providing asynchronous video content with regular phone check-ins to facilitate participation [40,41], 4 being synchronous group-based programs (2 video-based only [42,43], 1 providing mixed options [44], and 1 music group providing recorded sessions for later review by participants unable to attend [45]).

There were 2 synchronous video-based programs targeting caregivers, primarily aimed at enhancing their caregiving skills, such as symptom evaluation and management [46], as well as providing professional consultation, referral, and psychological support [47]. In total, 3 additional synchronous telehealth interventions were found to provide teleconsultation and social support to dyads of people living with dementia and their caregivers. One program involved connecting dyads with neurologists for teleconsultation via phone or video [48], whereas the other 2 programs organized group-based activities for dyads using videoconferencing platforms [49,50]. The smallest sample was from a case study that focused on only 1 person living with dementia, and the largest sample was from a caregiver survey study that included 130 respondents.

An overview of the 24 articles is provided in Multimedia Appendix 1. The reviewed articles were categorized based on the country in which the research was conducted and arranged alphabetically by country name. We extracted and reorganized information from each study into the following 4 a priori themes of interest: study design decisions, efficacy outcomes of telehealth interventions, perceived experiences of people living with dementia and caregivers, and barriers to accessing and using telehealth services. Textbox 1 provides a list of the subthemes under the 4 major themes.

**Table 1:** Descriptive characteristics of reviewed studies (n=24).

Characteristics			Studies, n (%)
**Research country**			
	Argentina	1 (4)	
	Brazil	2 (8)	
	Canada	1 (4)	
	China	1 (4)	
	Ireland	1 (4)	
	Italy	4 (17)	
	New Zealand	2 (8)	
	Spain	1 (4)	
	United Kingdom	5 (21)	
	United States	6 (25)	
**Research type**			
	Qualitative	14 (58)	
	Quantitative	10 (42)	
**Research focus**			
	Efficacy	5 (21)	
	Feasibility	10 (42)	
	Experience	9 (38)	
**Research sample**			
	People living with dementia only	6 (25)	
	Caregivers only	3 (13)	
	People living with dementia-caregiver dyads	13 (54)	
	Professional providers (physicians and intervention facilitators)	2 (8)	
**Telehealth type**			
	Routine medical care (medical visits and prescription refills)	6 (25)	
	Treatment^a^, rehabilitation, or intervention for people living with dementia only	13 (54)	
	Training^b^, support, or intervention for caregiver only	2 (8)	
	Telehealth to both people living with dementia and caregiver^c^	3 (13)	
**Delivery method**			
	Telephone only	2 (8)	
	Video only	15 (63)	
	Mixed (either-or and both)	7 (29)	

^a^Structured and time-limited cognitive, behavioral, and emotional treatment programs designed specifically for people living with dementia, such as cognitive stimulation, music therapy, and speech and language therapy.

^b^Structured and time-limited training programs designed for family caregivers to help them provide home care to people living with dementia, such as physical examinations, blood tests, and infusion management.

^c^Teleconsultation, remote peer support, and internet-based social activities that aim to facilitate social engagement and provide social support for people living with dementia and family caregivers.

**List of themes and subthemes.**

Study design characteristics

- Trade-offs between sample diversity and participant’s engagement in technology
- Choosing the right service delivery platform to increase accessibility and usability
- Putting in place safeguards to improve data collection and quality
- Improving participants’ engagement and retention

Efficacy outcomes of telehealth services

- Significant outcomes of people living with dementia
- Significant caregiver outcomes
- Studies with nonsignificant findings

Perceived experience of people living with dementia and caregivers

- Satisfaction with telehealth services
- Perceived personal benefits
- Perceived social benefits

Barriers to accessing and using telehealth services

- Individual factors
- Factors related to accessibility
- Telehealth environmental factors

### Theme 1: Study Design Characteristics

#### Overview

Telehealth research is faced with many challenges, such as how to ensure telehealth access for people who rarely use remote or internet-based services and ensure the efficacy or effectiveness of the services. These challenges are aggravated by the disruption of any in-person communications during the pandemic. This scoping review identified several study design characteristics corresponding to such challenges.

#### Subtheme 1a: Trade-offs Between Sample Diversity and Participant’s Engagement in Technology

In total, 5 studies used purposeful sampling to increase the representation of the participants from various backgrounds. For example, many older participants (late life), members of racial and ethnic minorities, people living in rural areas, and families with low and middle incomes were recruited [30,35,50]. For older people with hearing issues, Di Lorito et al [37] developed user guides to help participants navigate speakerphones. Kalicki et al [31] reported that community-dwelling older people may require more attention than those at residential facilities because the latter typically have better access to and support for technology at their facilities.

In contrast, it is noteworthy that socioeconomic characteristics were related to users’ access to telehealth services. An analysis of primary survey data revealed that older adults with low levels of education tended to be less accepting of telehealth services [27]. Some reviewed studies developed eligibility criteria to ensure participants’ internet access and service engagement and to reduce attrition rates. However, such criteria may inadvertently lower the sample diversity and representation of these studies by omitting potential participants who would face the greatest challenges in accessing telehealth technology. For instance, 2 articles excluded older adults who did not have electronic devices or had no caregivers and people with physical disabilities to ensure the success of the internet connection [35,36]. Moreover, 3 other articles used convenience samples with internet access and expertise from larger parent projects already in the field [34,47,50].

#### Subtheme 1b: Choosing the Right Service Delivery Platform to Increase Accessibility and Usability

Choosing a service delivery platform and mode in which users are accustomed can lessen the burden of learning and adaptation. Several researchers have attempted to enhance people living with dementia and caregivers’ accessibility and usability of telehealth services. First, 2 studies in Brazil used the country’s most popular instant messaging app (ie, WhatsApp) for medical consultation [27,35]. Moreover, 4 studies allowed participants to choose their preferred communication method with their physicians via telephone or video [28,30,37,48]. Weiss et al [39] implemented a 2-step approach wherein patients were first contacted via telephone and then transferred to a video call. Other steps included providing initial training, how-to instructions, and enrolling caregivers to help set up internet connections [38,41,43,47].

#### Subtheme 1c: Putting in Place Safeguards to Improve Data Collection and Quality

The COVID-19 pandemic necessitated the rapid transition of in-person programs to telehealth services, which required researchers to adopt various strategies to improve the quality and success of remote implementations. First, the researchers modified the facilitator guides and project protocols to accommodate the attributes of remote and internet-based services. For instance, several studies have sought advice from various stakeholders (eg, people living with dementia, caregivers, service providers, professionals, and clinical staff) on how to modify the protocol to best suit participant needs [42,44,46]. Telehealth program facilitators had the authority to adapt the content of interventions with supervisor’s weekly reviews to ensure that the adaptation was appropriate [44,45,50]. To increase the thoroughness and objectivity of the analysis, blind assessors were integrated into the data collection, and various data sources were used: quantitative, qualitative, and documentation review [32,34]. In addition, some privacy issues emerged: providers were concerned that patients were being less forthright when asked questions because the caregiver was present during the encounter. As a result, some providers asked the second person to leave the room for a short period when discussing sensitive topics [30].

#### Subtheme 1d: Improving Participants’ Engagement and Retention

Participants in telehealth interventions experienced benefits, such as enriched content, inclusive community, and individualized evaluation and support, which contributed to increased engagement and retention rates. For instance, a group music therapy program recorded every session and posted it on YouTube so that people who did not attend could view the session later [45]. To capture participants’ attention, 2 cognitive intervention studies for people living with dementia used visual and auditory components, such as pictures, music, and videos [41,42]. Support group meetings were organized to promote communication between participants and the facilitator [44,47,50]. Other studies included routine emotional screening or wellness assessments in addition to interventions that increased participant retention rates and identify any emergent psychological issues as soon as possible to provide support or referral [30,34,40,46].

### Theme 2: Efficacy Outcomes of Telehealth Services

#### Overview

Among the 5 articles that examined the efficacy of telehealth interventions for people living with dementia or caregivers, 2 offered cognitive teleinterventions for people living with dementia [33,34]; 2 offered integrated interventions that addressed social connectedness, physical health, psychological well-being, and cognitive stimulation for people living with dementia [36,40]; and 1 offered a teleconsultation for people with frontotemporal dementia and their caregivers [48]. Overall, 5 studies reported significant efficacy outcomes: 2 studies on people living with dementia and 3 studies on caregiver outcomes.

#### Subtheme 2a: Significant Outcomes of People Living With Dementia

Panerai et al [34] provided a telephone-based cognitive intervention for people living with dementia in Italy and found that the intervention group (n=14) outperformed the nontreatment group (n=13) on almost all outcomes after 1 month of training, including reduced behavioral and psychiatric symptoms and increased cognitive performance. Lai et al [36] from Hong Kong compared the effectiveness of telephone-based (n=30) and video-based teleinterventions (n=30) and found that the video-based intervention was more successful at preventing cognitive decline and enhancing the quality of life of people living with dementia.

#### Subtheme 2b: Significant Caregiver Outcomes

Despite the 2 studies by Panerai et al [34] and Lai et al [36] only provided interventions to people living with dementia, they also queried caregivers of people living with dementia in the assessment to comprehend the mechanisms by which interventions affected people living with dementia-caregiver dyads [48]. The findings of both studies revealed beneficial effects of teleinterventions on caregivers, including diminished burden and perceived distress, as well as improved self-rated health and self-efficacy [34,36]. Some caregivers said that their relationships with people living with dementia had improved [36]. In addition, Capozzo et al [48] measured caregivers’ self-efficacy and found significant improvement after receiving teleconsultation, which is probably because of their improved knowledge of the health status of people living with dementia [48].

#### Subtheme 2c: Studies With Nonsignificant Findings

In total, 2 studies did not report significant findings. Dorman et al [33] in Argentina offered people living with dementia a 2-month cognitive teleintervention, but no significant difference was found in all people living with dementia outcomes (ie, activities of daily living, instrumental activities of daily living, depression, and anxiety) between the intervention and control groups. The researchers explained that this may be related to weak analytic power, a nonrandomized controlled trial research design, and low intervention efficacy. Goodman-Casanova et al [40] in Spain adapted a television-based intervention for people living with dementia by adding modules of COVID-19 knowledge, health and social support resources, and physical health. Interestingly, 58 (64%) participants reported that too much COVID-19 information increased their anxiety and decreased their interest in continuing to use other intervention modules. Nevertheless, no statistical difference was found between participants who complained about the adapted content and those who did not complain in all outcomes (ie, anxiety, psychological well-being, and sleep quality).

### Theme 3: Perceived Experience of People Living With Dementia and Caregivers

#### Subtheme 3a: Satisfaction With Telehealth Services

In total, 19 articles reported the feasibility and acceptability of telehealth services by evaluating people living with dementia and caregivers’ subjective experiences of use. Overall, both people living with dementia and caregivers were satisfied with the use of telehealth services and reported a willingness to continue using them in the future [27,35,39,45,47,48]*.* Lima et al [35] found that 90% of people living with dementia were satisfied with teleconsultation with physicians. Moreira-Constantin et al [27] and Capozzo et al [48], respectively, reported that caregiver satisfaction with telehealth services for people living with dementia was 67% and 81%. Gately et al [38] reported that 14 people living with dementia-caregiver dyads’ satisfaction with telehealth programs ranged from 4.5 to 4.9 on a 5-point scale [38].

#### Subtheme 3b: Perceived Personal Benefits

People living with dementia reported 3 personal benefits from receiving telehealth services at home. First, receiving consultation or intervention at home can make people living with dementia feel more comfortable, reduce their anxiety about unfamiliar settings, and enhance the impact of intervention or treatment [45]. Second, videoconferencing platforms enable therapists to use visual and auditory materials to draw the attention of people living with dementia for better communication [37,41]. Third, compared with in-person services, some people living with dementia believe that facilitators would pay more attention to and respond more quickly to their emotional needs when using telehealth services [50]. Caregivers also reported 3 perceived personal benefits of using telehealth programs, including having an outlet to express frustration, saving travel time and financial costs of visiting clinics or treatment centers, gaining more respite time when people living with dementia meet with physicians at home [43,47,50], and having the opportunity to communicate with physicians and neurologists to learn about dementia and caregiving [43,44,50].

#### Subtheme 3c: Perceived Social Benefits

According to the reviewed studies, telehealth programs were found to provide 2 major social benefits to people living with dementia and caregivers during the COVID-19 pandemic: maintaining contact with health care professionals, family members, and communities; and making new friends [32,43,50]. Such social benefits then promote participants’ engagement in telehealth programs, thus facilitating their successful implementation of the programs. For instance, Quail et al [41] in the United Kingdom found that teleintervention improved the cognitive function and emotional regulation of people living with dementia, allowing them to communicate with family members more effectively. Participants in 2 other cognitive and well-being teleinterventions said that making friends from various linguistic, cultural, and historical backgrounds through group-based activities kept them interested and involved in the programs [44,50].

### Theme 4: Barriers to Accessing and Using Telehealth Services

#### Overview

Researchers have summarized the difficulties in the adoption of telehealth programs among people living with dementia and caregivers during the COVID-19 pandemic, which can be classified into three primary aspects: (1) individual factors, (2) factors related to accessibility, and (3) environmental factors.

#### Subtheme 4a: Individual Factors

Health status has a considerable impact on how people living with dementia and their caregivers use telehealth programs. Owing to the decline in cognitive function, people living with dementia frequently struggle to focus and communicate with physicians or therapists on the web [35,40,44]. According to research, those with severe cognitive impairment as well as other disabilities, such as vision and hearing issues, are less likely to connect to and use telehealth services than those with early-stage dementia [30,31,49]. Second, because of the long history of using in-person services, many participants disliked telehealth [30,44,50]. Some people even believe that telehealth is not a true medical service and are unwilling to pay for it [27,46]. The third factor is participants’ concerns about their privacy and safety, which prevent open communication on many sensitive topics [41,46]. Some caregivers are also wary of internet-based programs because it is difficult to determine the authenticity of web-based information and people living with dementia are more susceptible to scams and cyberbullying [43].

#### Subtheme 4b: Factors Related to Accessibility

In total, 15 studies mentioned that the digital divide and inequality are widely present among older adults, posing challenges for the transition of dementia dyads to telehealth during the COVID-19 pandemic. Arighi et al [29] reported that the domestic broadband network coverage in Italy for households where people living with dementia reside alone was only 34%, which is even lower in low- and middle-income countries. In addition, many dementia dyads have low digital literacy, such as not knowing how to install or use web-based medical platforms [28,37,51]. Despite 3 studies offering pretraining for caregivers to assist with internet connection, most participants still struggled with the transition to telehealth, especially people living with dementia who have no available caregivers [41,42,48]. One quantitative analysis revealed that when both telephone and video options were offered, most participants opted to rely on telephone calls [48].

#### Subtheme 4c: Telehealth Environmental Factors

Several studies have indicated that the telehealth environment has restrictions compared with in-person settings, which may limit the advantages of telehealth. First, remote communication may miss nonverbal cues. For example, providers cannot capture the facial expressions and body language of participants when talking over the phone, leading to less effective communication than video-based and face-to-face interactions [28,30]. Second, remote communication may have restricted participants’ engagement. Lee et al [45] and O’Connor et al [47] reported that some participants hardly engaged in video-based group activities because only 1 person could speak at a time and they were unable to talk individually, as each statement had to be addressed to the entire group [45,47]. Third, medical professionals raised concerns about accurately assessing patients’ performance and environmental risks during rehabilitation sessions, whether over the phone or via video [31]. Finally, some interventions and treatments, such as clinical tests that require the use of specialized equipment, and physical training and rehabilitation that require close physical supervision by therapists, cannot be delivered using telehealth [35,46].

## Discussion

### Principal Findings

Telehealth has gained considerable attention during the COVID-19 pandemic, and reimbursement policies in health care settings have increased access to remote modes of care delivery. Telehealth has the potential to mitigate care concerns and social isolation among people living with dementia and their family caregivers. Our scoping review examined the feasibility, acceptability, and efficacy of telehealth programs for people living with dementia and caregivers during the COVID-19 pandemic. Overall, our study findings indicate that telehealth programs have the potential to meet the medical, psychological, and social demands and preferences of people living with dementia-caregiver dyads during the pandemic, yet more research is needed to ascertain the conduct and quality of these programs.

### Key Contributions of the Reviewed Studies

This review revealed that telehealth services were crucial in assisting people living with dementia-caregiver dyads during the COVID-19 pandemic. Telehealth has the potential to prevent deterioration of cognition and other psychiatric symptoms in people living with dementia and can also help relieve caregivers’ perceived distress, give them respite time, provide information, and improve the quality of their interactions with people living with dementia [34,36,47,48]. Most participants had favorable opinions regarding telehealth programs. Although true, it is unknown if the positive perceptions were due in part to the fact that telehealth was their only option or the ability to interact with other participants and health care providers, which may have had a protective effect on their psychological health and quality of life [44,50]. For participants who live in rural areas and are remote from health care providers, most of them thought that telehealth services were as effective as in-person visits because of reduced commuting time [28,37]. The positive attitude of participants was also indicated by their high adherence to telehealth visits because some participants expressed a desire to continue participating in group-based activities as they had developed positive relationships with other participants [33,41].

In terms of mode of delivery, video calls were found to be more efficient than phone calls because service providers reported making more accurate judgments based on participants’ facial expressions and body movements [36]. Nevertheless, the digital divide was a barrier for most people living with dementia to accept telehealth services [28,29,37,51]. Caregivers, especially more tech-savvy individuals, can greatly improve the accessibility of telehealth services among people living with dementia [29]. More investment is needed in the future to help dementia care dyads resolve the digital divide, adapt to mandatory lockdowns that may occur in future pandemics, and reduce their impact on mental health and quality of life.

### Limitations of the Reviewed Studies

Despite the emerging positive findings from these studies, this study has some limitations. The studies’ research designs were mostly in the pilot phase of development, which poses questions about the efficacy of the interventions. Although some studies have set up nonrandomized control groups [33,34,36], none have used randomized control designs. Most studies had small sample sizes, and none established a follow-up assessment of the sustainability of the intervention effects [33-35,38].

Other limitations include the lack of description of study participants’ characteristics, and some excluded participants if they were not able to have consistent internet connections [27,35,36]. Although this scoping review found studies from 10 different countries, many participant characteristics, such as race, ethnicity, socioeconomic status, and the stages of dementia in people living with dementia, are still underreported. As a result, these findings have limited implications for dementia dyads from underrepresented groups.

The details of the implementation procedure have not yet been fully disclosed. Only 38% (9/24) of studies mentioned strategies to ensure participant engagement and retention [30,34,40-42,44-46,50]. In total, 2 studies used low participant dropout rates to document that participants demonstrated high engagement in telehealth programs [33,48]. In addition, some studies that examined the feasibility of shifting in-person programs to the web did not report participants’ transitional experience, the success rate of the transition, and the strategies adopted to help participants quickly adjust to the remote service pattern [49,50].

Most of the reviewed studies (20/24, 83%) were conducted in high-income countries (ie, the United States, Canada, Ireland, Italy, Spain, the United Kingdom, and New Zealand). Only 4 studies were conducted in countries in the upper middle–income tier: Argentina, Brazil, and China. These 4 studies were all conducted in large cities where people have relatively higher incomes and better access to medical resources [27,33,35,36]. However, many low- and middle-income regions still lack basic internet services and convenient transportation and have been hit hard during the COVID-19 pandemic [52].

Finally, some participants expressed concerns about safety and privacy while using telehealth services [41,43,46], but no studies have reported using any appropriate cybersecurity measures to protect personal health information [53].

### Future Work

On the basis of the findings of this review, there are several potential directions for future research. Adopting a dyadic perspective to interview people living with dementia and caregivers improves the quality of studies by providing a more holistic view of telehealth services and their benefits.

The content of telehealth programs needs to be continuously optimized and improved. For example, there are differences between teleservice and in-person visits in terms of the type and depth of communication and interaction between participants and service providers. Using the same content and protocols of in-person programs for the web-based programs may affect the quality of services and participants’ experiences. For example, using a wearable device to monitor a patient’s heart rate and blood pressure during a telehealth visit can enhance the assessment of the participant’s health during the visit, thus aiding better patient care [54]. Although the restriction of social activities can easily affect the mood of people living with dementia and caregivers, reviewed studies have paid little attention to participants’ emotional health and needs. Telehealth services should consider adding emotional support modules, such as emotional health screening, psychological counseling, and mental health service referral [34].

With the increase in the rates of COVID-19 vaccination and the development of effective treatments, it is expected that more people will return to living in the postpandemic world [55]. However, history indicates that there will always be new pandemics or other disasters. To better respond to the challenges that may arise in the future, policy makers and medical and social service providers should continue to target at-risk groups, especially those with neurocognitive impairments, such as dementia and family caregivers, who are the backbone of long-term services and support.

### Study Limitations

Although this scoping review was conducted following PRISMA-ScR methodology, there are still some limitations. First, the COVID-19 pandemic is a time-sensitive topic and dependent on rapid medical and technological advances. What may be true at the beginning of the pandemic may be less so in later phases. The performance of these telehealth programs for people living with dementia-caregiver dyads during the pandemic may not be thoroughly represented by the 24 reviewed studies. Second, the review selected articles published in English, which may have limited the understanding of research published in other languages. Third, the exclusion of preprint servers from the search may have led to the omission of relevant and timely information on ongoing studies. However, including preprint servers requires careful evaluation based on comprehensive criteria, which can be time consuming and disputes inducing, requiring additional resources to address.

### Conclusions

This scoping review identified 24 studies that examined the feasibility, acceptability, and efficacy of telehealth services among people living with dementia and their caregivers during the period of COVID-19 pandemic. The reviewed studies suggest that telehealth services have the potential to address the challenges faced by people living with dementia and their caregivers, such as improving their mental health, maintaining quality of life, and preventing functional decline. Both people living with dementia and caregivers reported an interest in telehealth services and were willing to continue using such services during the postpandemic era. Future research should adopt a dyadic perspective to better understand the needs of both people living with dementia and their caregivers, which can benefit the design and effectiveness of telehealth programs. Collaborative efforts by stakeholders at various levels should focus on closing the digital divide facing people living with dementia-caregiver dyads, to promote their accessibility toward telehealth programs, and to better prepare for potential future disasters.

## Appendix

Multimedia Appendix 1 Summary table of the reviewed articles (n=24).

## Abbreviations

PRISMA: Preferred Reporting Items for Systematic Reviews and Meta-Analyses
PRISMA-ScR: Preferred Reporting Items for Systematic Reviews and Meta-Analyses extension for Scoping Reviews

## References

[1] Pierce M (2020). The impact of COVID-19 on people who use and provide Long-Term Care in Ireland and mitigating measures. LTCcovid.org, International Long-Term Care Policy Network, CPEC-LSE.

[2] Gilstrap L, Zhou W, Alsan M, Nanda A, Skinner JS (2022). Trends in mortality rates among medicare enrollees with Alzheimer disease and related dementias before and during the early phase of the COVID-19 pandemic. JAMA Neurol.

[3] Carpinelli Mazzi M, Iavarone A, Musella C, De Luca M, de Vita D, Branciforte S, Coppola A, Scarpa R, Raimondo S, Sorrentino S, Lualdi F, Postiglione A (2020). Time of isolation, education and gender influence the psychological outcome during COVID-19 lockdown in caregivers of patients with dementia. Eur Geriatr Med.

[4] Altieri M, Santangelo G (2021). The psychological impact of COVID-19 pandemic and lockdown on caregivers of people with dementia. Am J Geriatr Psychiatry.

[5] Borges-Machado F, Barros D, Ribeiro Ó, Carvalho J (2020). The effects of COVID-19 home confinement in dementia care: physical and cognitive decline, severe neuropsychiatric symptoms and increased caregiving burden. Am J Alzheimers Dis Other Demen.

[6] Meshkat S, Salimi A, Joshaghanian A, Sedighi S, Sedighi S, Aghamollaii V (2020). Chronic neurological diseases and COVID-19: associations and considerations. Transl Neurosci.

[7] Calo WA, Murray A, Francis E, Bermudez M, Kraschnewski J (2020). Reaching the Hispanic community about COVID-19 through existing chronic disease prevention programs. Prev Chronic Dis.

[8] Wang H, Li T, Barbarino P, Gauthier S, Brodaty H, Molinuevo JL, Xie H, Sun Y, Yu E, Tang Y, Weidner W, Yu X (2020). Dementia care during COVID-19. Lancet.

[9] Bacsu JR, O'Connell ME, Webster C, Poole L, Wighton MB, Sivananthan S (2021). A scoping review of COVID-19 experiences of people living with dementia. Can J Public Health.

[10] Suzuki M, Hotta M, Nagase A, Yamamoto Y, Hirakawa N, Satake Y, Nagata Y, Suehiro T, Kanemoto H, Yoshiyama K, Mori E, Hashimoto M, Ikeda M (2020). The behavioral pattern of patients with frontotemporal dementia during the COVID-19 pandemic. Int Psychogeriatr.

[11] Migliaccio R, Bouzigues A (2020). Dementia and COVID-19 lockdown: more than a double blow for patients and caregivers. Alzheimers Dis Rep.

[12] Killen A, Olsen K, McKeith I, Thomas A, O'Brien JT, Donaghy P, Taylor J (2020). The challenges of COVID-19 for people with dementia with Lewy bodies and family caregivers. Int J Geriatr Psychiatry.

[13] Boutoleau-Bretonnière C, Pouclet-Courtemanche H, Gillet A, Bernard A, Deruet A, Gouraud I, Lamy E, Mazoué A, Rocher L, Bretonnière C, El Haj M (2020). Impact of confinement on the burden of caregivers of patients with the behavioral variant of frontotemporal dementia and Alzheimer disease during the COVID-19 crisis in France. Dement Geriatr Cogn Dis Extra.

[14] Hwang Y, Connell LM, Rajpara AR, Hodgson NA (2021). Impact of COVID-19 on dementia caregivers and factors associated with their anxiety symptoms. Am J Alzheimers Dis Other Demen.

[15] Beheshti L, Kalankesh LR, Doshmangir L, Farahbakhsh M (2022). Telehealth in primary health care: a scoping review of the literature. Perspect Health Inf Manag.

[16] Rajasekaran K (2020). Access to telemedicine-are we doing all that we can during the COVID-19 pandemic?. Otolaryngol Head Neck Surg.

[17] Chiang L, Chen W, Dai Y, Ho Y (2012). The effectiveness of telehealth care on caregiver burden, mastery of stress, and family function among family caregivers of heart failure patients: a quasi-experimental study. Int J Nurs Stud.

[18] Kitsiou S, Paré G, Jaana M, Gerber B (2017). Effectiveness of mHealth interventions for patients with diabetes: an overview of systematic reviews. PLoS One.

[19] Dang S, Gomez-Orozco CA, van Zuilen MH, Levis S (2018). Providing dementia consultations to veterans using clinical video telehealth: results from a clinical demonstration project. Telemed J E Health.

[20] Gately ME, Trudeau SA, Moo LR (2019). In-home video telehealth for dementia management: implications for rehabilitation. Curr Geriatr Rep.

[21] Koma W, Cubanski J, Neuman T (2021). Medicare and telehealth: coverage and use during the COVID-19 pandemic and options for the future. KFF.

[22] Zhong NS, Zheng BJ, Li YM, Poon L, Xie ZH, Chan KH, Li PH, Tan SY, Chang Q, Xie JP, Liu XQ, Xu J, Li DX, Yuen KY, Peiris J, Guan Y (2003). Epidemiology and cause of severe acute respiratory syndrome (SARS) in Guangdong, People's Republic of China, in February, 2003. Lancet.

[23] Langford AT, Solid CA, Scott E, Lad M, Maayan E, Williams SK, Seixas AA (2019). Mobile phone ownership, health apps, and tablet use in US adults with a self-reported history of hypertension: cross-sectional study. JMIR Mhealth Uhealth.

[24] Tricco AC, Lillie E, Zarin W, O'Brien KK, Colquhoun H, Levac D, Moher D, Peters MD, Horsley T, Weeks L, Hempel S, Akl EA, Chang C, McGowan J, Stewart L, Hartling L, Aldcroft A, Wilson MG, Garritty C, Lewin S, Godfrey CM, Macdonald MT, Langlois EV, Soares-Weiser K, Moriarty J, Clifford T, Tunçalp Ö, Straus SE (2018). PRISMA extension for scoping reviews (PRISMA-ScR): checklist and explanation. Ann Intern Med.

[25] Kellermeyer L, Harnke B, Knight S (2018). Covidence and Rayyan. J Medical Library Assoc.

[26] Clarke V, Braun V (2018). Using thematic analysis in counselling and psychotherapy research: a critical reflection. Couns Psychother Res.

[27] Moreira-Constantin B, Carpen-Padovani G, Cordeiro-Gaede AV, Chamma-Coelho A, Martínez-Souza R K, Nisihara R (2022). Telemedicine in the monitoring of patients with dementia: a Brazilians caregivers's perspective. Rev Neurol.

[28] Roach P, Zwiers A, Cox E, Fischer K, Charlton A, Josephson C, Patten S, Seitz D, Ismail Z, Smith E (2021). Understanding the impact of the COVID-19 pandemic on well-being and virtual care for people living with dementia and care partners living in the community. Dementia (London).

[29] Arighi A, Fumagalli GG, Carandini T, Pietroboni AM, De Riz MA, Galimberti D, Scarpini E (2021). Facing the digital divide into a dementia clinic during COVID-19 pandemic: caregiver age matters. Neurol Sci.

[30] Tuijt R, Rait G, Frost R, Wilcock J, Manthorpe J, Walters K (2021). Remote primary care consultations for people living with dementia during the COVID-19 pandemic: experiences of people living with dementia and their carers. Br J Gen Pract.

[31] Kalicki AV, Moody KA, Franzosa E, Gliatto PM, Ornstein KA (2021). Barriers to telehealth access among homebound older adults. J Am Geriatr Soc.

[32] Macchi ZA, Ayele R, Dini M, Lamira J, Katz M, Pantilat SZ, Jones J, Kluger BM (2021). Lessons from the COVID-19 pandemic for improving outpatient neuropalliative care: a qualitative study of patient and caregiver perspectives. Palliat Med.

[33] Dorman G, Alvarez Dengra A, Fiorini A, Failla B, Vallejos F, Pontello N, Roca M, Bustin J (2020). Experience and results with a telehealth treatment program in patients with cognitive disorders during the COVID-19 pandemic. Int J Geriatr Psychiatry.

[34] Panerai S, Raggi A, Tasca D, Musso S, Gelardi D, Prestianni G, Catania V, Muratore S, Ferri R (2021). Telephone-based reality orientation therapy for patients with dementia: a pilot study during the COVID-19 outbreak. Am J Occup Ther.

[35] Lima DP, Queiroz IB, Carneiro AH, Pereira DA, Castro CS, Viana-Júnior AB, Nogueira CB, Coelho Filho JM, Lôbo RR, Roriz-Filho JD, Braga-Neto P (2022). Feasibility indicators of telemedicine for patients with dementia in a public hospital in Northeast Brazil during the COVID-19 pandemic. PLoS One.

[36] Lai FH, Yan EW, Yu KK, Tsui W, Chan DT, Yee BK (2020). The protective impact of telemedicine on persons with dementia and their caregivers during the COVID-19 pandemic. Am J Geriatr Psychiatry.

[37] Di Lorito C, Duff C, Rogers C, Tuxworth J, Bell J, Fothergill R, Wilkinson L, Bosco A, Howe L, O'Brien R, Godfrey M, Dunlop M, van der Wardt V, Booth V, Logan P, Cowley A, Harwood RH (2021). Tele-rehabilitation for people with dementia during the COVID-19 pandemic: a case-study from England. Int J Environ Res Public Health.

[38] Gately ME, Muccini S, Eggleston BA, McLaren JE (2022). Program evaluation of my life, my story: virtual storytelling in the COVID-19 age. Clin Gerontol.

[39] Weiss EF, Malik R, Santos T, Ceide M, Cohen J, Verghese J, Zwerling JL (2021). Telehealth for the cognitively impaired older adult and their caregivers: lessons from a coordinated approach. Neurodegener Dis Manag.

[40] Goodman-Casanova JM, Dura-Perez E, Guzman-Parra J, Cuesta-Vargas A, Mayoral-Cleries F (2020). Telehealth home support during COVID-19 confinement for community-dwelling older adults with mild cognitive impairment or mild dementia: survey study. J Med Internet Res.

[41] Quail Z, Bolton L, Massey K (2021). Digital delivery of non-pharmacological intervention programmes for people living with dementia during the COVID-19 pandemic. BMJ Case Rep.

[42] Cheung G, Peri K (2021). Challenges to dementia care during COVID-19: innovations in remote delivery of group Cognitive Stimulation Therapy. Aging Ment Health.

[43] Peri K, Balmer D, Cheung G (2023). The experiences of carers in supporting a person living with dementia to participate in virtual cognitive stimulation therapy during the COVID-19 pandemic. Aging Ment Health.

[44] Cooper C, Mansour H, Carter C, Rapaport P, Morgan-Trimmer S, Marchant NL, Poppe M, Higgs P, Brierley J, Solomon N, Budgett J, Bird M, Walters K, Barber J, Wenborn J, Lang IA, Huntley J, Ritchie K, Kales HC, Brodaty H, Aguirre E, Betz A, Palomo M (2021). Social connectedness and dementia prevention: pilot of the APPLE-Tree video-call intervention during the Covid-19 pandemic. Dementia (London).

[45] Lee S, O’Neill D, Moss H (2021). Dementia-inclusive group-singing online during COVID-19: a qualitative exploration. Nordic J Music Therapy.

[46] Marinello R, Brunetti E, Luppi C, Bianca D, Tibaldi V, Isaia G, Bo M (2021). Telemedicine-assisted care of an older patient with COVID-19 and dementia: bridging the gap between hospital and home. Aging Clin Exp Res.

[47] O'Connor MK, Nicholson R, Epstein C, Donley T, Salant R, Nguyen AH, Shirk S, Stevenson E, Mittelman MS (2023). Telehealth support for dementia caregivers during the COVID-19 pandemic: lessons learned from the NYU family support program. Am J Geriatr Psychiatry.

[48] Capozzo R, Zoccolella S, Frisullo M, Barone R, Dell’Abate Mt, Barulli M, Musio M, Accogli M, Logroscino G (2020). Telemedicine for delivery of care in frontotemporal lobar degeneration during COVID-19 pandemic: results from Southern Italy. J Alzheimer's Disease.

[49] Giebel C, Cannon J, Hanna K, Butchard S, Eley R, Gaughan A, Komuravelli A, Shenton J, Callaghan S, Tetlow H, Limbert S, Whittington R, Rogers C, Rajagopal M, Ward K, Shaw L, Corcoran R, Bennett K, Gabbay M (2021). Impact of COVID-19 related social support service closures on people with dementia and unpaid carers: a qualitative study. Aging Ment Health.

[50] Masoud SS, Meyer KN, Martin Sweet L, Prado PJ, White CL (2021). "We don't feel so alone": a qualitative study of virtual memory cafés to support social connectedness among individuals living with dementia and care partners during COVID-19. Front Public Health.

[51] Gedde M, Husebo B, Erdal A, Puaschitz N, Vislapuu M, Angeles R, Berge L (2021). Access to and interest in assistive technology for home-dwelling people with dementia during the COVID-19 pandemic (PAN.DEM). Int Rev Psychiatry.

[52] Leite H, Hodgkinson I, Gruber T (2020). New development: ‘healing at a distance’—telemedicine and COVID-19. Public Money Manage.

[53] O'Herrin JK, Fost N, Kudsk KA (2004). Health Insurance Portability Accountability Act (HIPAA) regulations: effect on medical record research. Ann Surg.

[54] Materia FT, Smyth JM (2021). Acceptability of intervention design factors in mHealth intervention research: experimental factorial study. JMIR Mhealth Uhealth.

[55] (2022). The end of the COVID-19 pandemic is in sight: WHO. UN News.

