# Supplementary material for: The Use of Telehealth Among People Living With Dementia-Caregiver Dyads During the COVID-19 Pandemic: Scoping Review
Source: J Med Internet Res. 2023 May 25;25:e45045. doi: 10.2196/45045 (PMC10251224; doi:10.2196/45045)
Supplement: Multimedia Appendix 1 [file jmir_v25i1e45045_app1.pdf]

Multimedia Appendix 1. Summary table of reviewed articles (n = 24)

Author, date, country	Type of Research	Aim	Sample	Service	Access Consideration	Major Findings
Dorman et al., 2020 Argentina	Quantitative (two group intervention, not RCT)	To test the effectiveness of a telehealth program that aims to reduce the risk of ADL decline and the onset or worsening of behavioral and psychological symptoms in people living with dementia during the COVID-19 pandemic.	Intervention group: 27 people living with dementia & 19 caregivers;<br>Control group: 11 people living with dementia & 7 caregivers.	A telehealth treatment programs (TTP) consists of 1/2/3 30-min phone calls/week between therapists and the people living with dementia, accompanied with caregivers.<br>Treatment includes cognitive/music/occupational/speech and language therapy.	Not found	High adherence rate (100%) within the initial 2-month treatment;<br>No significant difference was found in all outcomes (ADL, IADL, depression, anxiety) between intervention and control groups.
Lima et al., 2022 Brazil	Quantitative (single group intervention)	To describe feasibility of telemedicine tools, including patient recruitment, attendance, discomfort, satisfaction, travel time/cost savings.	85 people living with dementia who used face-to-face service at the study hospital during the previous 12 months.	WhatsApp-based remote medical consultation for people living with dementia.<br>Intervention includes text and video treatment, health promotion, drug prescription, diagnostic tests, and referrals.<br>One time similar to face-to-face consultation.	Send instructions in prior (e.g., choose strong WIFI, have caregiver/family to assist, have test results and medications at hand);<br>Exclude who have no communication technology, and have no caregiver to assist.	Recruitment rate: 85.5% (89/104);<br>Attendance rate: 97.7% (87/89);<br>Completion rate: 97.7% (85/87);<br>Discomfort rate: 9.4% (8/85);<br>Satisfaction rate: 90% accept the telemedicine approach, 45% believe it's effective;<br>Saved 233 min and 11 USD on travel each time.
Moreira-Constantin et al., 2022 Brazil	Quantitative (primary survey data analysis)	To analyze the preferences of caregivers of people living with dementia on the use of telemedicine in monitoring patients.	130 caregivers of people living with dementia.	General telemedicine service	Not found	98% have access to internet, 72% familiar with telemedicine, 33% have used telemedicine, 27% would like to pay for tele-consultation;<br>40% believe telemedicine is the future, 47% think it does not diminish relationship, and 67% believe it has the same validity of face-to-face service.

Roach et al., 2020 Canada	Qualitative (individual interview)	To determine the extent to which the COVID-19 pandemic and related public health measures impacted experiences of health care.	2 PLWD and 20 care partners/family members.	General telemedicine service	Not found	Advantages:<br>Both doctors and participants feel less rushed;<br>Care partners can be more candid if the patients were not in the call;<br>Disadvantages:<br>Less opportunity for care partner to be frank if the patient was present;<br>Technology barriers; Non-verbal information is hard to communicate remotely.
Lai et al., 2020 China	Quantitative (two groups intervention, not RCT)	To test the effectiveness of telehealth delivered via video-conferencing platforms in addition to routine telephone calls on PLWD (65−80 years old) in home care and their spousal caregivers over a period of social distancing.	Intervention group:<br>30 PLWD & 30 caregivers;<br>Control group: 30 PLWD & 30 caregivers.	Weekly 30min telephone covering topics and information related to older adults' well-being of community living, focusing on health aging, psychological needs, and physical well-being;<br>Intervention group received additional 30 min video service (Zoom, WhatsApp, Facetime).	Only include those who have caregivers (to provide consent and help set up the connection);<br>Videoconferencing app was pre-installed by caregivers before intervention.	PLWD: Video-based telehealth can better prevent decline of cognitive function and quality of life; memory and behavioral problems not differed between 2 groups;<br>Caregiver: health, burden, and self-efficacy all get worse in control group, while improved in intervention group;<br>The video service facilitate interaction between care-recipient and caregivers.
Lee et al., 2021 Ireland	Qualitative (individual interview)	To capture how COVID-19 has impacted dementia-inclusive choirs and singing groups in Ireland (facilitators' perception, adaptation, response)?	12 facilitators of dementia-inclusive singing groups.	Online music therapy activities (singing groups, choirs) on Zoom, WhatsApp, Facebook, YouTube, and other virtual platforms.	Provide asynchronous resources for participation/practice at any time;<br>Community health worker provide tech support and encourage PLWD to engage.	Positive:<br>Online tech provide accessibility and inclusivity, online session keeps PLWD life structured during lockdown, keep PLWD socially connected, give caregiver respite, music inspires online social connection.<br>Negative:<br>In-person experience is irreplaceable, tech can be barriers to users.

Arighi et al., 2021<br>Italy	Quantitative (primary survey data analysis)	To describe the digital divide of PLWD contacted by telemedicine during the COVID-19 pandemic and to understand which factors can influence tele-medicine successfulness.	108 PLWD.	General telemedicine service (video-based).	Instructions to connect were sent by mail;<br>Caregivers were contacted by phone if PLWD cannot connect by self.	74 (68.5%) successfully connected, 34 failed due to lack of devices and low tech savviness.<br>PLWD with young caregiver are more likely to connect successfully;<br>Successfulness also increase with time.
Capozzo et al., 2020<br>Italy	Quantitative (primary survey data analysis)	To evaluate whether the assessment of PLWD using telemedicine is feasible and acceptable to patients and caregivers in the era of the COVID-19 pandemic.	4 Frontotemporal dementia (FTD) patients and 28 FTD caregivers.	Teleconsultation by neurologist to patients or caregivers (60-90 min tele/cell-phone).	Video option was provided (but refused by all due to lack of devices and low familiarity).	21 participants (66%) completed satisfactory survey;<br>Most (81%) are satisfied with tele-medicine;<br>88% are satisfied when interacting with neurologist;<br>89.5% agree that tele-consultation help receive service easily at home (70% live far away);<br>90% like to continue this way in the future.
Panerai et al., 2021<br>Italy	Quantitative (two groups intervention, not RCT)	To test effectiveness of telephone-based reality orientation therapy (T–ROT) among patients with major neurocognitive disorders (NCDs) and also whether T–ROT can relieve the burden of distress among caregivers.	Intervention group: 14 dementia care dyads;<br>Control group: 13 dementia care dyads.	Telephone-based Reality Orientation Therapy (T-ROT): stimulation of orientation to time, place, and person to promote a sense of control over the environment.<br>2 assessment sessions + 10 intervention sessions over 4 weeks (3 sessions /week)	Not found	T–ROT significantly outperformed nontreatment;<br>Intervention group showed decreased symptoms, depression, increase in cognition.<br>Caregivers showed lower burden and distress.
Marinello et al., 2021<br>Italy	Qualitative (single case report)	To describe one atypical COVID case that were successfully managed at home thanks to caregivers and telemedicine service.	A 91-year-old older woman with dementia and atypical COVID-19 symptoms.	Caregivers at home received tele-instruction (video-based training and monitoring) from physicians at hospital to perform treatment on patients;<br>Twice a day.	Not found	The caregiver with high motivation provided good treatment to the patient under the guidance and supervision of physicians from hospitals;<br>Patient restored and continued using telemedicine until discharge.

Cheung & Peri, 2021 New Zealand	Qualitative (a study case report)	To describe how tech-based virtual Cognitive Stimulation Therapy (vCST) was used to deliver intervention for PLWD during COVID-19.	10 groups, no details of participants report.	14 sessions (2 session/week) for 7 weeks;<br>45 min per session;<br>6-8 persons per group.	Trainers provide guidance on moving service to online;<br>Tech support (e.g., pre-training, check connection);<br>Choose Zoom as platform since most facilitators are familiar with it.	10 vCST successfully implemented, no other details reported.
Peri et al., 2022 New Zealand	Qualitative (individual interview)	To explore the carer's roles and experiences of supporting PLWD when attending vCST.	12 carers (38 contacted, 31.6% response rate) recruited via 5 organizations.	14 sessions (2 session/week) for 7 weeks;<br>45 min per session;<br>6-8 persons per group.	Pre-training to carers and PLWD on how to access via Zoom;<br>Pre-survey on the IT device ownership.	Carers help with tech set-up and feel positive about learning and improving their tech knowledge;<br>Carers enjoyed the social contact with facilitators, respite time, and sense of connection during vCST;<br>Carers feel both improvement and challenge on their relationship with PLWD.
Goodman-Casanova et al., 2020 Spain	Quantitative (primary survey data analysis, post intervention)	To study the effects of a television-based assistive integrated technology (TV-AssistDem).	93 participants recruited from a large project (47 intervention, 46 control); 21 interviewed caregivers.	TV-AssistDem: a television-based service for PLWD to support service on health and well-being, social connectedness, and cognitive stimulation;<br>COVID-19 adapted contents include official information, care measures.	Participants recruited from a large project, thus device has been set-up.	58 (64%) feel accessed too much information regarding COVID-19; 29 (31%) request more;<br>No difference in any outcomes between intervention and control groups.
Cooper et al., 2021 UK	Qualitative (single group intervention)	To investigate how acceptable and feasible the intervention was to deliver in practice, in the context of the pandemic.	12 PLWD;<br>10 finished all interventions;<br>8 completed post-intervention assessment.	APPLE_Tree: video-call, group-based cognitive well-being intervention.<br>10 sessions and every week has: 1-hour group video call (structured), 0.5-hour group video call (tea break, unstructured), and 0.5-hour phone call with 1 facilitator to check and set goals.	Add a facilitator to help adapt remote delivery (internet connection or other tech issues);<br>Gave training/practice before intervention on how to use Zoom (telephone support);	83% sessions finished, 64% tea break attendance rate, 82% goals achieved; Overall delivery fidelity is 86%;<br>Although there were tensions for some participants, they enjoyed social aspects of the groups.

Giebel et al., 2021 UK	Qualitative (individual interview)	To explore the effects of COVID-19 related social care and social support service closures on the lives of PLWD and unpaid carers.	42 Carers, 8 PLWD	Peer support groups shift to remote delivery on Zoom	Not found	Only a few shifted online;<br>Feel cannot make up for the face-to-face session;<br>Many lost contact due to tech limit or health conditions.
Di Lorito et al., 2021 UK	Qualitative (a study case report)	To identify participants that video delivery worked for; to highlight its benefits and its challenges.	5 dementia care dyads (10 persons); and 5 therapists.	Q Health: England-approved video patient consultation platform.	Provide options of phone or video call;<br>Consent was taken orally;<br>Protocol developed in collaboration with PLWD;<br>Use speakerphone to assure hear and respond to the questions.	Tele-rehabilitation works better for older adults with better physical health (lower risk of falling), digital ability, with caregiver aside, and with good rapport with the therapist.
Quail et al., 2021 UK	Qualitative (single case report)	To describe the transition from in-person dementia therapy and group-based therapeutic activity delivery to digital delivery.	A man at late 60s referred by his therapist with a diagnosis of AD.	Multimodal programme of interventions aim of attaining meaningful engagement in activities (cognitive stimulation, reminiscence therapy, music therapy, physical exercise, reality orientation, validation therapy);<br>2-3 times per week.	Award-winning YouTube video freely available to explain therapy;<br>Family got involved to help set up infrastructure.	The case person enjoyed improvements in cognition and engagement in activities, which indicates the program prevent the cognitive deterioration due to COVID-19 distress or dementia progression.
Tuijt et al., 2021 UK	Qualitative (individual interview)	To explore experiences of remote health care during the COVID-19 pandemic in the community.	30 PLWD and 31 carers.	General telemedicine service (remote primary care consultations).	Purposive sampling tries to reflect a variety of ages, types of dementia, sex, ethnicity, and rurality;<br>Gave options of phone or video call.	Advantages: frequent phone-call check, useful information; most prefer remote consultations;<br>Disadvantages: PLWD has concern on carers' management of all conversations with professionals, low tech literacy, hearing or memory problems, hard to describe symptoms on phone, hard to keep appointment by self.

Gately et al., 2022<br>USA	Quantitative (single group intervention)	To evaluate the experience of Veteran dementia dyads on a telehealth program (MLMS).	14 Veteran-caregiver dyads	1-time Veteran-centric tele-intervention (my life my story, MLMS) via teleconferencing, phone, or a combination. Caregivers were involved to support Veterans. Quantitative experience assessment was conducted in follow-up interviews.	Assisted with technology set-up before interview (e.g., sending links).	11 used teleconferencing 3 used telephone only;<br>Caregivers helped with tech set-up;<br>Tech experience: most indicated positive experience with scores ranges from 4.5 to 4.9;<br>Interview experience: most ratings are positive ranges from 4.3 to 4.4.
Kalicki et al., 2021<br>USA	Quantitative (primary survey data analysis)	To understand existing video-based telehealth usage and capability among older patients.	16 physician providers, served 1000 (193 congregate housing residents who have tech access were excluded) patients with video-based telehealth since April 2020.	General video-based telehealth services (home-based primary care)	N/A;<br>The aim of the study is to identify accessibility of patients.	563 (65%) had no tech access;<br>153 cannot interact over internet by self;<br>274 need help with internet;<br>78 do not have caregiver to assist;<br>Physicians have limited knowledge on tech and economic barriers of patients.
Macchi et al., 2021<br>USA	Qualitative (individual interview)	To describe the impact of COVID-19 on patients living with chronic, neurodegenerative disease and their caregivers.	108 patients and 90 caregivers.	General telemedicine services	Not found	Positive: telecommunications help participants connect with healthcare services and family/friends;<br>Negative: telehealth is considered subpar to in-person services, not a long-term solution, and cannot supplant pre-COVID levels of care.
Masoud et al., 2021<br>USA	Qualitative (individual interview)	To describe the experiences of individuals living with dementia and family care partners who regularly attend Memory Cafés, and explores how these gatherings affect perceptions of social connectedness.	5 PLWD and 12 care partners.	Memory café: group-based activities allow PLWD and caregiver to socialize, focusing on ability still remains, and enjoy life out of caregiving responsibility.	Open to non-community resident;<br>Hosted in both English and Spanish.	2 in-person only, 9 virtual only, and 6 both.<br>Advantages: respite time, identify life possibilities, connect with community, inclusivity, diversity, additional value added (cognitive stimulation, health education, resource sharing, helping others).

O'Connor et al., 2022<br>USA	Qualitative (individual interview)	To understand how dementia caregivers experienced online support services after switching to videoconferencing during the COVID-19 pandemic.	10 dementia caregivers (convenience sample from the FSP database)	The NYU Langone ADRD Family Support Program (FSP) provides multiple caregiver support services: comprehensive evaluation, psycho-education, respite care, consultation, and professional referrals.<br>All the programs shifted online due to the pandemic.	Not found	Dementia caregivers felt supported by the online programs: outlet to express frustration, understand the situation, connection with social workers;<br>Some felt more connected via the online support groups, some others felt the relationship diminished due to video telehealth format;<br>Participants' suggestions: more multi-media content and informal venue to connect are needed to foster engagement and connections.
Weiss et al., 2021<br>USA	Qualitative (a study case report)	To demonstrate a unique approach to reaching culturally diverse and vulnerable population using telehealth, and share some of the lessons learned as a result of early difficulties.	85 PLWD.	CACARRE televisit model: introduction, review of health records, treatment, caregiver stress evaluation, advance care planning, emergency planning, respite care,	Allow telephone or live video to build relationship, then move to safe video platform;<br>Approach dyads through multidisciplinary collaboration.	Factors relate to no-show:<br>female, Black, high school graduates;<br>Most feel positive, helpful; caregiver feel all concerns addressed; many hope to return to in-person but maintain televisit for follow-up;<br>Can conduct real-time and accurate assessment;<br>More inclusive and accessible.
